# The Effect of Coatings on Cutting Force in Turning of C45 Steel

**DOI:** 10.3390/ma15020590

**Published:** 2022-01-13

**Authors:** Jaroslav Kovalčík, Petr Mašek, Jan Malý, Pavel Kožmín, Jiří Syrovátka

**Affiliations:** 1 Department of Production Machines and Equipment, Faculty of Mechanical Engineering, Czech Technical University in Prague, 128 00 Prague, Czech Republic; p.masek@rcmt.cvut.cz (P.M.); j.maly@rcmt.cvut.cz (J.M.); 2 Hofmeister, s.r.o., 301 00 Plzeň, Czech Republic; kozmin@hofmeister.cz (P.K.); syrovatka@hofmeister.cz (J.S.)

**Keywords:** turning, coating, carbon steel, modelling, cutting force

## Abstract

This article focuses on the development of a mathematical model of a cutting force that is applicable for coated and uncoated cutting tool inserts and aims to enable more accurate calculation of the cutting force. Two common PVD coatings, AlTiN and TiAlCrN, were used. Firstly, a mathematical model of the cutting force based on the specific cutting force and cutting area is proposed. This mathematical model considers the cutting speed and coating correction factor as well as the real cutting edge geometry, i.e., it includes both the straight and rounded parts of the cutting edge. For this proposed model, material constants for C45 steel, which was machined with uncoated inserts, were obtained. Before determining an equation for a coating correction factor and implementing it into the model, experimental cutting force data for coated and uncoated inserts were compared using a paired *t*-test. The result was that the difference between them was statistically significant. Their percentage difference was found to be up to 4%. The correction factor equation that was obtained and implemented into the mathematical model was applied to compare the calculated and experimental data of the coated inserts, also using a paired *t*-test. The result was that the difference between them was insignificant. Moreover, their percentage difference was found to be up to 0.6%.

## 1. Introduction

A coating provides a physical barrier between a cutting tool and the workpiece material, which protects the cutting tool against heat, abrasion, adhesion and the chemical effects of the close environment [1]. Coatings can have different chemical compositions based on ceramic compounds, carbides, nitrides, diamond-like materials or diamonds, etc. These chemical layers are often combined to improve the protection of the cutting tool edge. The coating also changes the cutting tool’s microgeometry in terms of the surface roughness on the rake and clearance face as well as the roundness of the cutting edge due to the non-zero thickness of the coating layer [2]. Sivam measured the non-zero effect of coatings on the forces, tool wear and quality of machined surfaces during titanium alloy machining through grey relational analysis [3]. He focused on the minimisation of a complex function with many outcomes including surface roughness, cutting force, tool wear and roundness. Differences in the cutting force and surface roughness of coated and uncoated tools has previously been measured [4]. Jindal investigated various coatings made through physical vapour deposition (PVD) [5]. He focused on the turning of an Inconel 718 sample and proved that the cutting tool composition had a significant influence on cutting tool wear. Fernandez-Abia presented the same result when using a different coating made through PVD during turning of stainless steel [6]. Venkantesh compared PVD single-layer and CVD (chemical vapour deposition) triple-layer coatings [7]. The cutting wear of the PVD coating was approximately 14% higher than that of the CVD coating, but the measured cutting forces were similar or only insignificantly higher for the PVD coating. Wang researched the difference in cutting force when turning an AISI S1020 steel bar using uncoated and CVD-coated inserts [8]. He found that the average difference was 7% for one type of CVD coating and 3% for the other type of CVD coating. Kulkarni [9] and Kamely [10] also found cutting force differences for various coatings. Kulkarni investigated this issue when turning an AISI 314 steel bar. In this case, the coating change caused a difference in the cutting force in the range of approximately 30–100 N depending on the cutting velocity for PVD coatings. Kamely [10] researched this issue when turning an AISI D2 bar. In this case, the difference was approximately 10–40 N depending on the cutting velocity. The differences in this case were caused by microgeometric variations in the coatings. Thus, the various coatings should cause a change in the force during turning of a particular workpiece, and this is verified in this paper in comparison with the cutting force model. Instability can be observed by force measurement and needs to be avoided during machining for comparison of different coatings in terms of the forces [11].

This paper focused on cutting force prediction for various coatings. Cutting force models can be obtained by various methods such as the finite element method (FEM), neural networks (ANNs), mathematical–statistical methods (e.g., RMS and regression analysis), empirical models and mechanistic models [12]. The FEM is based on simplification of a real model by elements with defined properties and behaviours. If the properties of these elements are properly adjusted, a model can be used to predict cutting forces, chip formation or heat in the cutting zone. This method was used by Galanis, and when compared to the real cutting experiment, achieved less than a 12% error [13]. Work by Parihar [14] achieved results with a slightly higher error. Kumar predicted the instability of a machining process for coated and uncoated tools using 2D FEM [15]. The ANN method is based on “elemental neurons”, where each neuron has many inputs but only one output. The inputs are transformed by the specific transfer function. Kara examined the possibility of using this method on the orthogonal turning of a pipe with various coatings with only a 5% error [16]. Quasim obtained similar results for turning of AISI 52100 [17]. Mia showed that the RSM force model was more accurate than an ANN for untrained data from machining titanium alloy [18]. The RSM model was also used by Noordin for the turning of AISI 1045 with a force prediction error of less than 2.7% [19]. Kolar used his statistical model, including cutting tool geometry and tool wear parameters, to predict forces in slot milling operations with 90% accuracy [20]. Another way to predict cutting forces is to use an empirical cutting model. Cutting forces are measured in a given range of cutting parameters, and then an empirical equation is constructed. This type of force modelling has been used by Shalaby [21], Stachursky [22] and Mahmud [23]. The final cutting force model is a mechanistic method. The mechanistic model is based on the strong relationship of the uncut chip thickness and measured forces. This relationship can be expressed by a linear function [24,25] or by a power model [26,27]. The Kienzle formula is often used as an expression of the mechanistic model. The specific cutting forces must be obtained experimentally, usually during orthogonal machining as described by Popovic [28]. Bera described a method for obtaining cutting force coefficients [29]. The basic equation can be extended for other significant factors and their coefficients. Horváth [30] used an effective length for his model that included the tool nose radius. These force prediction methods are usually chosen for one specific cutting tool and its coating.

The differences between coated and uncoated cutting tool inserts are not negligible in terms of wear progress, friction, heat conduction, microgeometry, and many other parameters, and these differences may influence the cutting force during machining. The cutting force model should reflect this influence and consider a coating correction factor. In this paper, an improvement that increases the accuracy of the basic Kienzle formula is described. The main aims of this study were to show the necessity of including the coating effect in the mechanistic model and to point out how coatings influence cutting forces.

## 2. Cutting Force Model

As previously mentioned, a coating may affect forces in machining. Predicting forces for a coated insert using a mathematical model based on an uncoated cutting tool insert is likely to be insufficiently accurate. This part of study focuses on the design of a predictive cutting force model with a correction factor for the coating. This model is expected to be applicable for coatings commonly used for cutting workpiece materials made of steel. The proposed mathematical model will be applicable for cases where the cutting tool holder inclination angle is 0°.

### 2.1. Force Decomposition in Turning

**Figure 1:**
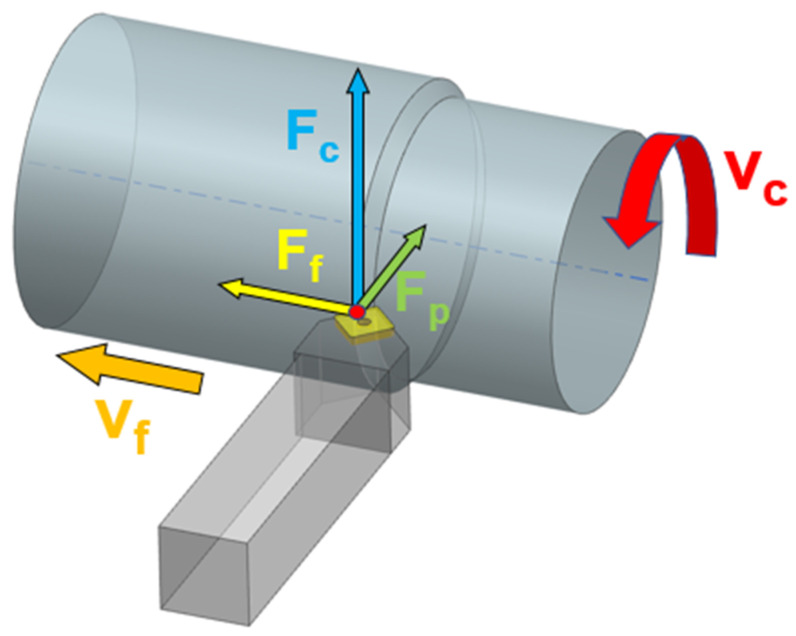
Force decomposition in longitudinal turning.

The main forces in turning include the cutting force ($F_{c}$), feed force ($F_{f}$) and passive force ($F_{p}$) as depicted in Figure 1.

The most important of these forces is the cutting force, which has the greatest effect on torque intensity. As mentioned in the introduction, this force may be calculated by various methods and principles.

### 2.2. Proposal of Methodology for Cutting Force Prediction

The total value of the cutting force is calculated by adding together the cutting force of the rounded ($F_{c_{1}}$) and straight ($F_{c_{2}}$) parts of the cutting edge; see Equation (1).

(1) $$F_{c}=F_{c_{1}}+F_{c_{2}}$$

Based on the literature review, we decided to use a cutting force modelling method based on the product of the specific cutting force ($k_{c}$) and undeformed chip area ($A_{D}$); see Equation (2).

(2) $$F_{c}=k_{c}\cdot A_{D}$$

To calculate the cutting force, the undeformed chip area and the specific cutting force have to be determined. The undeformed chip area is based on the product of the undeformed chip thickness ($h_{D}$) and the undeformed chip width ($b_{D}$); see Equation (3). Based on Reference [31], the specific cutting force for a workpiece made of C45 steel (as used in this article) is based on the impact of the chip thickness (the basic Kienzle formula) as well as the impact of the cutting speed; see Equation (4). In addition, this equation was modified by adding the coating correction factor, $K_{\mathrm{Coating}}$, which is determined experimentally here for some coatings commonly used for cutting workpiece materials made of steel.

(3) $$A_{D}=h_{D}\cdot b_{D}$$

(4) $$k_{c}=k_{c_{1.1}}\cdot h_{D}{}^{-m_{c}}\cdot {\left(\frac{v_{c}}{v_{c_{\mathrm{cp}}}}\right)}^{-n_{c}}\cdot K_{\mathrm{Coating}}$$

**Figure 2:**
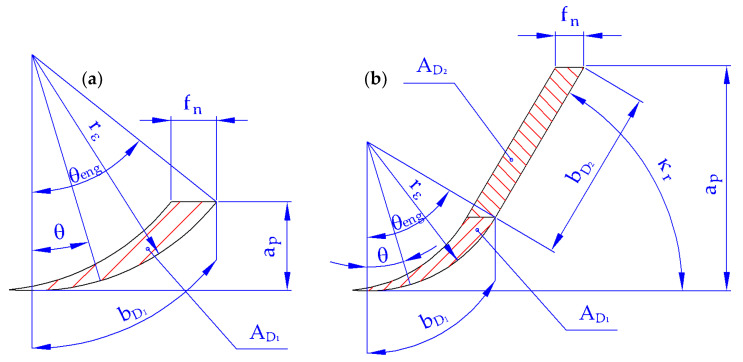
Cutting area when cutting: (**a**) with the rounded part of the cutting edge; (**b**) with the rounded as well as the straight part of the cutting edge.

The material constants for calculating the specific cutting force ($k_{c_{1.1}},{\;m}_{c},\;\mathrm{and}\;n_{c}$) have to be obtained for a specific workpiece material. The material constant $k_{c_{1.1}}$ is the specific cutting force for an undeformed chip area of 1 mm^2^ and for the cutting speed of the centre point ($v_{c_{\mathrm{cp}}}$). The centre point is the point in the centre of a designed plan of experiments. The material constant $m_{c}$ determines the effect of the undeformed chip thickness on the specific cutting force. The material constant $m_{v_{c}}$ determines the influence of the cutting speed on the specific cutting force. The undeformed chip thickness and the undeformed chip width are determined according to the schematics in Figure 2, which considers two cases. The first case is cutting a workpiece material using only the rounded part of the cutting edge, and the second case is cutting a workpiece material using both the rounded and straight parts of the cutting edge.

### 2.3. Rounded Part of the Cutting Edge

The undeformed chip thickness ($h_{D_{1}}$) of the rounded part of the cutting edge is calculated based on the feed per revolution ($f_{n}$) and the angle acting on the rounded part of the cutting edge (θ), which varies from 0° to reaching the engagement angle of the rounded part of the cutting edge ($\theta_{\mathrm{eng}}$); see Equation (5).

(5) $$h_{D_{1}}\left(\theta \right)=f_{n}\cdot \sin\theta$$

An element of the undeformed chip width of the rounded part of the cutting edge (${\mathrm{db}}_{D_{1}}$) is calculated based on the nose radius ($r_{\varepsilon }$) and the element of the angle acting on the rounded part of the cutting edge; see Equation (6).

(6) $${\mathrm{db}}_{D_{1}}=r_{\varepsilon }\cdot d\theta$$

The cutting force of the rounded part of the cutting edge ($F_{c_{1}}$) is obtained by substituting Equations (5) and (6) into Equation (2); see Equation (7). The integral given in this equation has no simple analytical solution.

(7) $$F_{c_{1}}=k_{c_{1.1}}\cdot f_{n}{}^{1-m_{c}}\cdot {\left(\frac{v_{c}}{v_{c_{\mathrm{cp}}}}\right)}^{-n_{c}}\cdot K_{\mathrm{Coating}}\cdot r_{\varepsilon }\cdot \int_{0}^{\theta_{\mathrm{eng}}}{\left(\sin\theta \right)}^{1-m_{c}}\;d\theta$$

The angle $\theta_{\mathrm{eng}}$ depends on whether only the rounded part of the cutting edge cuts the workpiece material (if the condition ${\;a}_{p}>r_{\varepsilon }\cdot \left(1-\cos\kappa_{r}\right)$ is met) or if both parts of the cutting edge (the rounded and straight parts) cut the workpiece material; see Equation (8).

(8) $$\begin{array}{cc} \theta_{\mathrm{eng}}= & \{\begin{array}{cc} \mathrm{arcos}\left(1-\frac{a_{p}}{r_{\varepsilon }}\right) & ,{\;a}_{p}>r_{\varepsilon }\cdot \left(1-\cos\kappa_{r}\right)\;\\ \kappa_{r} & ,{\;a}_{p}\leq r_{\varepsilon }\cdot \left(1-\cos\kappa_{r}\right) \end{array} \end{array}$$

### 2.4. Straight Part of the Cutting Edge

The straight part of the cutting edge cuts the workpiece only if the following condition is met: $a_{p}>r_{\varepsilon }\cdot \left(1-\cos\kappa_{r}\right)$; see Figure 2b. If this condition is not met, it means that only the rounded part of the cutting edge cuts the workpiece; see Figure 2a. In this case, the cutting force is zero.

The undeformed chip thickness of the straight part of the cutting edge ($h_{D_{2}}$) is calculated based on the feed per revolution ($f_{n}$) and the lead angle ($\kappa_{r}$); see Equation (9).

(9) $$h_{D_{2}}=f_{n}\cdot \sin\kappa_{r}$$

The undeformed chip width of the straight part of the cutting edge ($b_{D_{2}}$) is calculated for the case $\kappa_{r}\leq 90$° using Equation (10):

(10) $$b_{D_{2}}=\frac{a_{p}-r_{\varepsilon }\cdot \left(1-\cos\kappa_{r}\right)}{\sin\kappa_{r}}$$

The cutting force of the straight part of the cutting edge ($F_{c_{2}}$) is obtained by substituting Equations (9) and (10) into Equation (2); see Equation (11).

(11) $$F_{c_{2}}=k_{c_{1.1}}\cdot f_{n}{}^{1-m_{c}}\cdot {\left(\frac{v_{c}}{v_{c_{\mathrm{cp}}}}\right)}^{-n_{c}}\cdot K_{\mathrm{Coating}}\cdot \left[a_{p}-r_{\varepsilon }\cdot \left(1+\cos\kappa_{r}\right)\right]\cdot \;\;{\left(\sin\kappa_{r}\right)}^{-m_{c}}$$

### 2.5. Resultant Equations for the Cutting Force Calculation

If the condition $a_{p}\leq r_{\varepsilon }\cdot \left(1-\cos\kappa_{r}\right)$ is met, it means that both the straight and rounded parts of the cutting edge cut the workpiece material. In this case, the cutting force is calculated as the sum of the cutting forces of the straight and the rounded parts of the cutting edge; see Equation (12).

(12) $$F_{c}=F_{c_{1}}+F_{c_{2}}=k_{c_{1.1}}\cdot f_{n}{}^{1-m_{c}}\cdot {\left(\frac{v_{c}}{v_{c_{\mathrm{cp}}}}\right)}^{-n_{c}}\cdot K_{\mathrm{Coating}}\cdot r_{\varepsilon }\cdot \left[a_{p}-r_{\varepsilon }\cdot \left(1+\cos\kappa_{r}\right)\right]\;\cdot \;\;{\left(\sin\kappa_{r}\right)}^{-m_{c}}\cdot \int_{0}^{{}_{r}}{\left(\sin\theta \right)}^{1-m_{c}}\;d\theta$$

If the condition $a_{p}>r_{\varepsilon }\cdot \left(1-\cos\kappa_{r}\right)$ is met, it means that only the rounded part of the cutting edge cuts the workpiece material. In this case, the cutting force is calculated according to Equation (11).

## 3. Design of Experiments

### 3.1. Machine Tool

**Figure 3:**
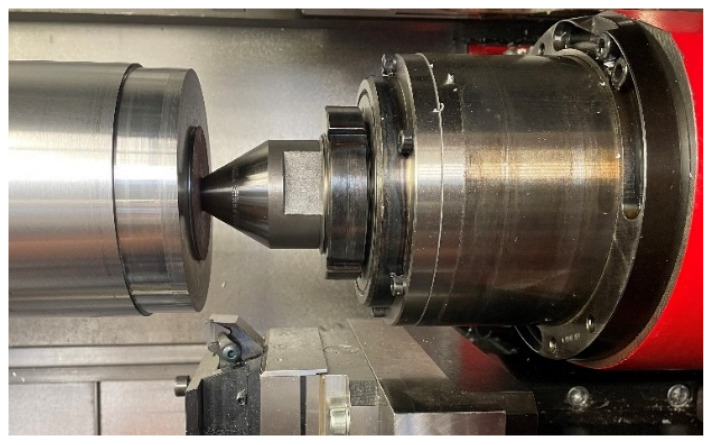
Experimental setup of the SP 430 SY 2 1100 CNC turning machine.

The experiments were carried out on an SP 430 SY 2 1100 CNC turning machine centre made by the Czech company Kovosvit MAS (Sezimovo Ústí, Czech Republic); see Figure 3. The maximum spindle power of the S1 mode was 28 kW, the maximum torque of the S1 mode was 1403 Nm, the nominal speed of the machine was 141 rpm and the maximum speed was 3150 rpm.

### 3.2. Cutting Tool

**Figure 4:**
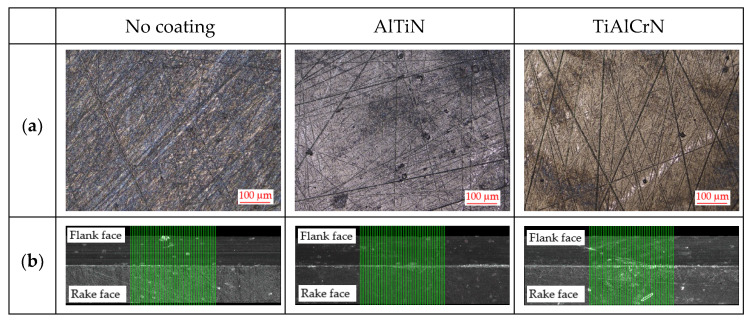
(**a**) Photos of the surfaces; (**b**) cutting edge radius measurements (10× magnified).

A CTGPR2525M3 cutting tool holder (Tungaloy Czech s.r.o., Brno, Czech Republic), with three types of TPGN160308 TH10 cutting tool inserts (Tungaloy Czech s.r.o., Brno, Czech Republic), without a chip breaker and with a nose radius of 0.8 mm, was used. The first type of insert had no coating, and the other two inserts were custom-coated with PVD coatings: AlTiN and TiAlCrN. These two coatings are commonly used for cutting workpiece materials made of steel. The photos of the surfaces of these three types of inserts were taken with an Alicona InfiniteFocus G5 (Alicona Imaging GmbH, Raaba/Graz, Austria), which is a highly accurate optical 3D measurement system; see Figure 4a. The lead angle (κ_r_) of the cutting tool holder was 90°, and the inclination angle (λ_s_) was 0°. The cutting edge radius (r) as well as the macrogeometry were measured with an Alicona InfiniteFocus G5. Each value for the cutting edge radius was evaluated as the average of 50 slices; see Figure 4b. As a matter of interest, all of the measured values of the cutting edge radius were smaller in our case than Bartoszuk found for similar coatings of custom-coated inserts [32]. A MarSurf LD 130 (Mahr, spol. s r.o., Proboštov, Czech Republic) was used to measure surface roughness values, which are shown in Table 1. The properties of both coated inserts are shown in Table 2.

### 3.3. Workpiece

The experiments were performed on a bar made of C45 carbon steel (KÖNIGFRANKSTAHL, s.r.o., Prague, Czech Republic). The hardness of this workpiece was measured 10 times with a KT-C hardness tester (NDT1 KRAFT Ltd., Prague, Czech Republic). The average hardness value was 192 ± 2 HB.

### 3.4. Measuring Devices

**Figure 5:**
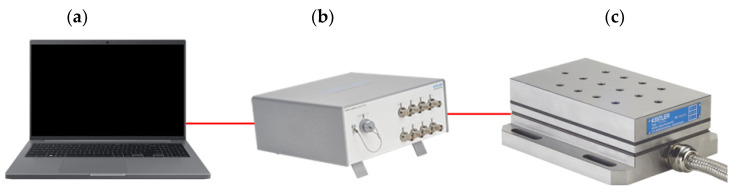
Measuring devices used to measure the force effects in turning: (**a**) PC; (**b**) LabAmp 5167A Laboratory Charge Amplifier DAQ; (**c**) 9257B dynamometer.

To measure the cutting forces in turning, a 9257B piezoelectric dynamometer (KISTLER, Prague, Czech Republic) with a 5167A laboratory charge amplifier (KISTLER, Prague, Czech Republic) with integrated data acquisition was used; see Figure 5.

### 3.5. Design of Experiments for Uncoated and Coated Cutting Tools

**Figure 6:**
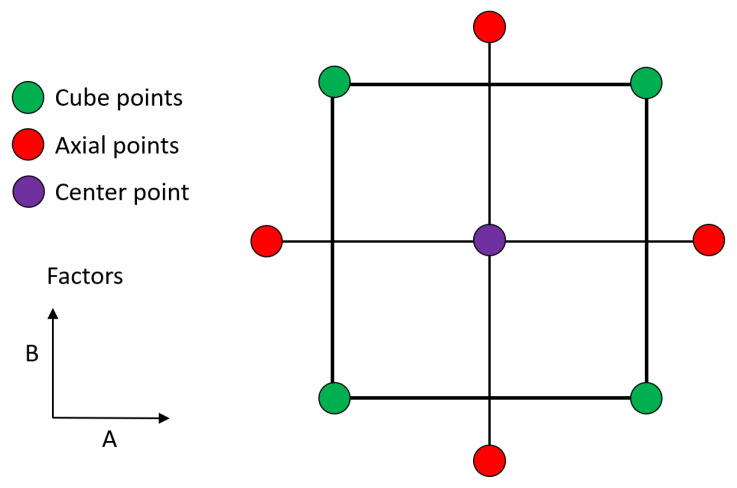
General scheme of central composite planning for two factors.

In order to design experiments for uncoated inserts to obtain the material constants for the mathematical model defined in the previous chapter, the central composite planning method for two factors was used. Figure 6 shows the general scheme of the planning method.

Factor A (no unit parameter) is characterised by the feed per revolutions (f_n_), and factor B (no unit parameter) is characterised by the cutting speed (v_c_).

The cutting conditions for the different levels of the central composite planning of the experiments are shown in Table 3, where α was set to 1.41421 by Minitab (Version: 20.1.1, Minitab Ltd., Coventry, UK).

As can be seen from Table 3, the axial depth of cut (a_p_) was constant and equal to 2 mm. Although this is the maximum value recommended by the manufacturer, it was set so the insert could cut the workpiece material largely by the straight part of the cutting edge. The feed per revolutions (f_n_) has five levels and was set with respect to the cube points in the range 0.12–0.20 mm. The cutting speed (v_c_) also has five levels and was set relative to the cube points in the range 120–200 m/min.

The design of experiments according to the central composite planning method for two factors with one centre point is shown in Table 4.

To obtain the correction factor of the coatings and also to verify the proposed mathematical model, the experiments for coated inserts were set by selecting the cube points and the centre point from Table 4.

## 4. Results and Discussions

### 4.1. Accuracy of the Proposed Mathematical Model for Uncoated Cutting Tools

The experiments for obtaining the material constants, which are shown in Table 4, were performed. Each experiment was measured two times (in some cases three times), and using these values, the average value and standard deviation were calculated; the results are shown in Table 5. Each cutting edge was used for three measurements, after which it was checked on a microscope to ensure that the cutting edge was not worn out.

The standard deviations of the evaluated cutting force values were in the range of 0.1–3.3 N. Based on these results, all the evaluated mean cutting force values were valid for obtaining the material constants.

To obtain the material constants, the specific cutting force values had to be calculated by dividing the cutting force from the experiment ($F_{c_{\exp}}$) by the calculated value of the undeformed chip area ($A_{D}$), which in this case is the total area, i.e. the area of the rounded and straight parts of the cutting edge; see Equation (13). The undeformed chip area of the rounded part of the cutting edge ($A_{D_{1}}$) is calculated based on Equations (5) and (6); see Equation (14). The undeformed chip area of the straight part of the cutting edge ($A_{D_{2}}$) is calculated based on Equations (9) and (10); see Equation (15). All the calculated values of the specific cutting force are presented in Table 6.

(13) $$k_{c}=\frac{F_{c_{\exp}}}{A_{D}}=\frac{F_{c_{\exp}}}{A_{D_{1}}+A_{D_{2}}}$$

(14) $$A_{D_{1}}=h_{D_{1}}\cdot b_{D_{1}}=f_{n}\cdot r_{\varepsilon }\cdot \int_{0}^{\kappa_{r}}\sin\theta \;d\theta =f_{n}\cdot r_{\varepsilon }\cdot \left[1-\cos\kappa_{r}\right]$$

(15) $$A_{D_{2}}=h_{D_{2}}\cdot b_{D_{2}}=f_{n}\cdot \left[a_{p}-r_{\varepsilon }\cdot \left(1-\cos\kappa_{r}\right)\right]$$

By using Minitab software, material constants were obtained based on the cutting speed and the calculated values of the undeformed chip thickness and the specific cutting force; see Equation (16). In the case of uncoated cutting tool inserts, the correction factor of the coating was 1.

(16) $$k_{c}=k_{c_{1.1}}\cdot h_{D}{}^{-m_{c}}\cdot {\left(\frac{v_{c}}{v_{c_{\mathrm{cp}}}}\right)}^{-n_{c}}=1317\cdot h_{D}{}^{-0.304}\cdot {\left(\frac{v_{c}}{160}\right)}^{-0.184}$$

The value of the determination index (*R*^2^) of the obtained mathematical model was 98.9%. Accordingly, the equation of the specific cutting force with the impact of the chip thickness and cutting speed with the obtained material constants was quite precise for prediction of the specific cutting force in the range of cutting conditions used for the experiments.

**Figure 7:**
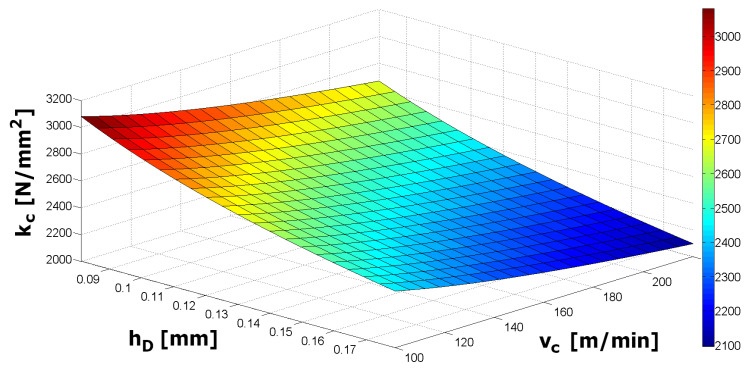
Dependence of the specific cutting force on undeformed chip thickness and cutting speed for uncoated inserts.

Figure 7 shows the dependence of the calculated values of the specific cutting force on the undeformed chip thickness (over the range of feeds per revolution from 0.10 to 0.22 mm) and the cutting speed (across the range of 100–220 m/min).

A paired *t*-test was carried out to determine whether there was a statistically significant difference between the experimental and calculated cutting force values; the results are given in Table 7. The null hypothesis was that there was no difference between them.

Since the *p*-value was much higher than the confidence level α = 0.05, the null hypothesis cannot be rejected and, therefore, it can be stated that there was not a statistically significant difference between the experimental and calculated cutting force values.

### 4.2. Comparing the Experimental Cutting Force Values of Uncoated and Coated Cutting Tools

The experimental cutting force values measured using the coated inserts (i.e., AlTiN and TiAlCrN), which were used to determine the coating correction factor and to verify the proposed mathematical model, are summarised in Table 8, along with the cutting forces measured by using uncoated inserts.

**Figure 8:**
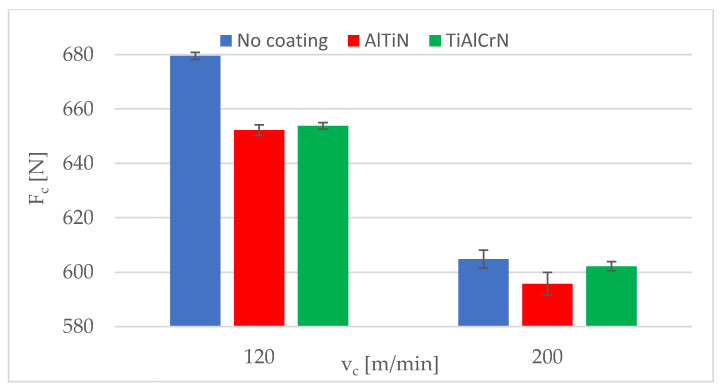
Experimental cutting force values for a feed per revolution of 0.12 mm.

**Figure 9:**
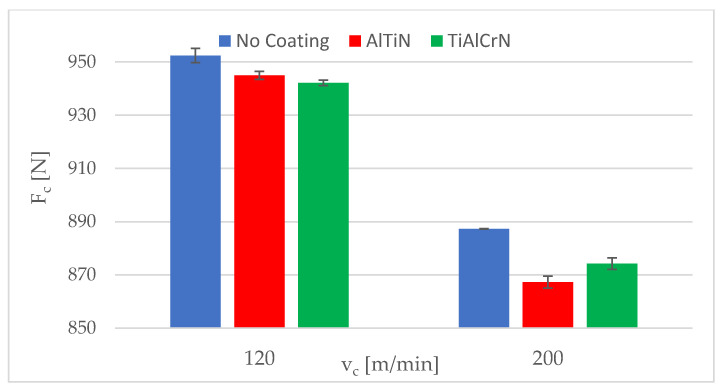
Experimental cutting force values for a feed per revolution of 0.20 mm.

The standard deviations of the evaluated mean cutting force values were in the range of 1.1–4.2 N for the AlTiN coating and 0.1–2.2 N for the TiAlCrN coating. Figure 8 and Figure 9 show the experimental cutting force values of the uncoated and coated inserts for a feed per revolution of 0.21 and 0.20 mm, respectively. As shown in Table 8 as well as in Figure 8 and Figure 9, the uncoated inserts had the highest cutting force values in all cases. There was only a minimal difference between the two coatings in terms of cutting force.

A paired *t*-test was carried out to determine whether there was a statistically significant difference between the cutting force values of the uncoated and coated cutting tool inserts as well as between the coatings themselves; the results are presented in Table 9. The null hypothesis was that there was no difference between them.

Since the *p*-value was below the confidence level α = 0.05, the null hypothesis was rejected in the comparison of the cutting force values of no coating and the AlTiN coating as well as in the comparison of the no coating and the TiAlCrN coating. This means that there was a statistically significant difference between these experimental cutting force data. In the comparison of the cutting force values of the AlTiN and the TiAlCrN coatings, the null hypothesis was not rejected, which means that there was not a statistically significant difference between the cutting force values.

### 4.3. Determining the Coating Correction Factor

Based on the conclusion in Section 4.2 that there was a statistically significant difference between no-coating and coating cutting force values, it was deemed necessary to find a coating correction factor (K_Coating_) that would be implemented into the proposed mathematical model; see Equation (4). This correction factor applies to each set of cutting conditions; it is calculated using the ratio between the cutting force determined from the proposed mathematical model, which was obtained for uncoated cutting tool inserts and the cutting force for a specific coating. The calculated correction factor values are shown in Table 10.

Based on Table 9, it was found that there was no statistically significant difference between the cutting force values when comparing the experimental cutting force values of the AlTiN and TiAlCrN coatings. Therefore, all of the correction factor values for both coatings were used to find a regression equation for the correction factor and for the analysis of variance. The linear regression coefficients are shown in Table 11.

Based on Table 11, the equation for calculating the coating correction factor is given by Equation (17).

(17) $$K_{\mathrm{Coating}}=0.944+0.195\cdot f_{n}+0.000134\cdot v_{c}$$

For the given linear regression (17), an analysis of variance (ANOVA) was performed; the results are presented in Table 12. It is obvious that the feed per tooth as well as the cutting speed were statistically significant parameters. In addition, since the *p*-value was higher than the significance level (α = 0.05), the null hypothesis cannot be rejected. This means that there was not enough evidence to conclude that there was a lack of fit in the linear regression model.

### 4.4. Accuracy of the Proposed Mathematical Model for Coated Cutting Tools

A statistically significant difference between no-coating and coating cutting force values is reported in Chapter 4.2. Therefore, a coating correction factor equation was found and implemented into the mathematical model. In this part of the study, the cutting force values calculated using the proposed mathematical model, which included the coating correction factor, were compared with the experimental cutting force values of the coated cutting tool inserts. All of these values are shown in Table 13.

A paired *t*-test was performed to determine whether there was a statistically significant difference between the calculated and experimental cutting force values; the results are presented in Table 14. The null hypothesis was that there was no difference between them.

Since the *p*-value was much higher than the confidence level α = 0.05, the null hypothesis cannot be rejected. This means that there was no statistically significant difference between the experimental and calculated cutting force data.

## 5. Conclusions

This study proposed a mathematical cutting force model that is applicable for cutting workpiece materials made of C45 steel and for coated and uncoated cutting tool inserts to enable more accurate calculation of the cutting force. Two commonly used PVD coatings, AlTiN and TiAlCrN, were used for the cutting workpiece materials. The key conclusions are summarised as follows:

- In the comparison of the experimental cutting force data for the uncoated and coated inserts, there was a statistically significant difference resulting from the paired *t*-test *p*-values (no coating–AlTiN: *p*-value = 0.011; no coating–TiAlCrN: *p*-value = 0.024), which were below the confidence level (α = 0.05); see Table 9. The percentage difference was found to be up to 4%;
- In the comparison of the experimental cutting force data for the two coated inserts, there was no statistically significant difference resulting from the paired *t*-test *p*-value (*p*-value = 0.392), which was above the confidence level (α = 0.05); see Table 9. This was despite the fact that the measured properties of the coatings were slightly different. The percentage difference was up to 1%;
- As there was no statistically significant difference between the two coated inserts, a linear regression was found for a coating correction factor that was valid for the two researched coatings, i.e., AlTiN and TiAlCrN. This regression included the impact of the feed per revolution as well as the cutting speed, which were statistically significant parameters according to the analysis of variance *p*-values (f_n_: *p*-value = 0.003; v_c_: *p*-value = 0.017), which were below the confidence level (α = 0.05); see Table 12;
- When the calculated cutting force data, which included the coating correction factor, were compared with the experimental data of the coated inserts, there was no statistically significant difference resulting from the paired *t*-test *p*-values (Model–AlTiN: *p*-value = 0.234; Model–TiAlCrN: *p*-value = 0.374), which were above the confidence level (α = 0.05); see Table 14. The percentage difference was found to be up to 0.6%.

## Figures and Tables

**Table 1:** Measured cutting tool geometry values.

Parameter	Unit	Symbol	No Coating	Coating	
				AlTiN	TiAlCrN
Cutting edge radius	μm	r	8.94 ± 0.67	10.45 ± 1.28	11.42 ± 0.58
Surface roughness	μm	Ra	0.13	0.16	0.24
Clearance angle	°	α_o_	11	11	11
Wedge angle	°	β_o_	79	79	79
Rake angle	°	γ_o_	0	0	0

**Table 2:** Properties of selected coated cutting tool inserts.

Property	Unit	Symbol	AlTiN	TiAlCrN
Coating thickness	μm	h	2.2	4.3
Hardness	GPa	H	23.8 ± 3.32	19.8 ± 1.9
Young’s modulus	GPa	E	685 ± 25.1	469 ± 21.2
Chemical composition	%	Ti	24.4 ± 0.14	13.3 ± 0.14
	%	Al	24 ± 0.03	14.8 ± 0.03
	%	Cr	-	24.3 ± 0.06
	%	N	51.6 ± 0.07	47.5 ± 0.27

**Table 3:** Cutting conditions for the different levels of the central composite planning.

Parameter	Unit	Symbol	Levels				
			−α	−1	0	+1	+α
Feed per revolution	mm	f_n_	0.103	0.12	0.16	0.20	0.217
Cutting speed	m/min	v_c_	103	120	160	200	217
Depth of cut	mm	a_p_	2				

**Table 4:** Design of experiments for uncoated CTI.

Type of Points	No Unit Parameters		Cutting Conditions	
	A	B	f_n_ (mm)	v_c_ (m/min)
Cube points	−1	−1	0.12	120
	+1	−1	0.20	120
	−1	+1	0.12	200
	+1	+1	0.20	200
Axial points	−α	0	0.103	160
	+α	0	0.217	160
	0	−α	0.16	103
	0	+α	0.16	217
Centre point	0	0	0.16	160

**Table 5:** Mean cutting force values with standard deviations.

Cutting Conditions		Results	
f_n_ (mm)	v_c_ (m/min)	F_c_ (N)	SD (N)
0.12	120	679.5	1.3
0.20	120	952.4	2.7
0.12	200	604.8	3.3
0.20	200	887.3	0.1
0.103	160	581.5	0.6
0.217	160	977.7	0.8
0.16	103	847.1	1.4
0.16	217	740.2	0.5
0.16	160	782.9	0.3

**Table 6:** Calculated specific cutting force values.

Cutting Conditions		Results		
f_n_ (mm)	v_c_ (m/min)	F_c exp_ (N)	A_D_ (mm^2^)	k_c_ (N/mm^2^)
0.12	120	679.5	0.240	2831
0.20	120	952.4	0.400	2381
0.12	200	604.8	0.240	2520
0.20	200	887.3	0.400	2218
0.103	160	581.5	0.206	2823
0.217	160	977.7	0.434	2253
0.16	103	547	0.320	2647
0.16	217	740.2	0.320	2313
0.16	160	782.9	0.320	2446

**Table 7:** Estimation for paired difference and test statistics.

Mean	SD	SE Mean	95% CI for μ_Difference	T-Value	*p*-Value
0.20	7.71	2.57	(−5.72; 6.13)	0.08	9.39 × 10^−1^

**Table 8:** Mean cutting force values with standard deviations.

Cutting Conditions		No Coating		AlTiN		TiAlCrN	
f_n_ (mm)	v_c_ (m/min)	F_c_ (N)	SD (N)	F_c_ (N)	SD (N)	F_c_ (N)	SD (N)
0.12	120	679.5	1.3	652.2	1.9	653.7	1.2
0.20	120	952.4	2.7	944.9	1.5	942.1	1.0
0.12	200	604.8	3.3	595.8	4.2	602.2	1.7
0.20	200	887.3	0.1	867.3	2.3	874.3	2.2
0.16	160	782.9	0.3	762.4	1.1	760.2	0.1

**Table 9:** Estimation for paired difference and test statistics.

Data to Compare	Mean	SD	SE Mean	95% CI for μ_Difference	T-Value	*p*-Value
No coating–AlTiN	16.86	8.39	3.75	(6.44; 27.28)	4.49	1.10 × 10^−2^
No coating–TiAlCrN	14.88	9.40	4.21	(3.20; 26.56)	3.54	2.40 × 10^−2^
AlTiN–TiAlCrN	−1.98	4.61	2.06	(−7.71; 3.75)	−0.96	39.20 × 10^−2^

**Table 10:** Mean cutting force values with standard deviations.

Cutting Conditions		F_c_ (N)			K_Coating_ (-)	
f_n_ (mm)	v_c_ (m/min)	Model	Experiment		AlTiN	TiAlCrN
			AlTiN	TiAlCrN		
0.12	120	662.3	652.2	653.7	0.985	0.987
0.20	120	945.1	944.9	942.1	1	0.997
0.12	200	602.9	595.8	602.2	0.988	0.999
0.20	200	860.3	867.3	874.3	1.008	1.016
0.16	160	767.4	762.4	760.2	0.993	0.991

**Table 11:** Linear regression coefficients.

Term	Coefficient	SE Coefficient	T-Value	*p*-Value
Constant	9.44 × 10^−1^	9.91 × 10^−3^	95.30	0.00
f_n_	1.95 × 10^−1^	4.32 × 10^−2^	4.50	0.30 × 10^−2^
v_c_	1.34 × 10^−4^	4.30 × 10^−5^	3.10	1.70 × 10^−2^

**Table 12:** Analysis of variance.

Source	DF	Adj SS	Adj MS	F-Value	*p*-Value
Regression	2	7.15 × 10^−4^	3.57 × 10^−4^	14.93	0.30 × 10^−2^
f_n_	1	4.85 × 10^−4^	4.85 × 10^−4^	20.24	0.30 × 10^−2^
v_c_	1	2.30 × 10^−4^	2.30 × 10^−4^	9.62	1.70 × 10^−2^
Error	7	1.68 × 10^−4^	0.24 × 10^−4^		
Lack of fit	2	0.67 × 10^−4^	0.34 × 10^−4^	1.68	27.70 × 10^−2^
Pure error	5	1.00 × 10^−4^	0.20 × 10^−4^		
Total	9	8.82 × 10^−4^			

**Table 13:** Calculated and experimental cutting force values of coated cutting tool inserts.

Cutting Conditions		F_c_ (N)		
f_n_ (mm)	v_c_ (m/min)	Model	Experiment	
			AlTiN	TiAlCrN
0.12	120	651.4	652.2	653.7
0.20	120	944.2	944.9	944.2
0.12	200	599.4	595.8	599.4
0.20	200	868.8	867.3	868.8
0.16	160	764.9	762.4	764.9

**Table 14:** Estimation for paired difference and test statistics.

Data to Compare	Mean	SD	SE Mean	95% CI for μ_Difference	T-Value	*p*-Value
Model–AlTiN	1.22	1.95	0.97	(−1.196; 3.636)	1.40	2.34 × 10^−1^
Model–TiAlCrN	−0.46	1.03	0.46	(−1.737; 0.817)	−1.00	3.74 × 10^−1^

## Data Availability

Data are contained within the article.

## Nomenclature

$\Delta F_{c}$ (%)	Percentage deviation between experimental and calculated cutting force values
$\alpha_{o}$ (°)	Clearance angle
$\beta_{o}$ (°)	Wedge angle
$\gamma_{o}$ (°)	Rake angle
θ (°)	Actual value of engagement angle of the rounded part of the cutting edge
$\theta_{\mathrm{eng}}$ (°)	Engagement angle of the rounded part of the cutting edge
$\kappa_{r}$ (°)	Lead angle
$\lambda_{s}$ (°)	Inclination angle
A (-)	No unit factor for DOE that corresponds to the feed per revolution
$A_{D}$ (mm^2^)	Undeformed chip area
$A_{D_{1}}$ (mm^2^)	Undeformed chip area of the rounded part of the cutting edge
$A_{D_{2}}$ (mm^2^)	Undeformed chip area of the straight part of the cutting edge
$a_{p}$ (mm)	Depth of cut
B (-)	No unit factor for DOE that corresponds to the cutting speed
$b_{D}$ (mm)	Undeformed chip width
$b_{D_{1}}$ (mm)	Undeformed chip width of the rounded part of the cutting edge
$b_{D_{2}}$ (mm)	Undeformed chip width of the straight part of the cutting edge
F (N)	Resultant force
$F_{c}$ (N)	Cutting force
$F_{c_{1}}$ (N)	Cutting force of the rounded part of the cutting edge
$F_{c_{2}}$ (N)	Cutting force of the straight part of the cutting edge
$F_{c_{\exp}}$ (N)	Cutting force evaluated from experiment
$F_{f}$ (N)	Feed force
$F_{p}$ (N)	Passive force
$f_{n}$ (mm)	Feed per revolution
$h_{D}$ (mm)	Undeformed chip thickness
$h_{D_{1}}$ (mm)	Undeformed chip of the rounded part of the cutting edge
$h_{D_{2}}$ (mm)	Undeformed chip thickness of the straight part of the cutting edge
$K_{\mathrm{Coating}}$ (-)	Correction factor of coating
$k_{c}$ (N/mm^2^)	Specific cutting force
$k_{c_{1}}$ (N/mm^2^)	Specific cutting force of the rounded part of the cutting edge
$k_{c_{2}}$ (N/mm^2^)	Specific cutting force of the straight part of the cutting edge
$k_{c_{1.1}}$ (N/mm^2^)	Specific cutting force for an undeformed chip area of 1 mm^2^
$m_{c}$ (-)	Empirical constant that indicates the impact of the chip thickness on the specific cutting force
$n_{c}$ (-)	Empirical constant that indicates the impact of the cutting speed on the specific cutting force
r (mm)	Cutting edge radius
$r_{\varepsilon }$ (mm)	Nose radius
SD (N)	Standard deviation of the cutting force
$v_{f}$ (mm/min)	Feed rate speed
$v_{c}$ (m/min)	Cutting speed
$v_{c_{\mathrm{cp}}}$ (m/min)	Cutting speed of the centre point

## References

[1] Leyendecker T., Lemmer O., Esser S., Ebberink J. (1991). The development of the PVD coating TiAlN as a commercial coating for cutting tools. Surf. Coat. Technol..

[2] Tobota D., Chechowski K., Wronska I., Letocha A., Miller T. (2013). The effects of the coating stripping process on regenerated tool cutting edges. J. Achiev. Mater. Manuf. Eng..

[3] Sivam S., Loganathan G., Saravanan K., RajendraKumar S. (2019). Outcome of the Coating Thickness on the Tool Act and Process Parameters When Dry Turning Ti–6Al–4V Alloy: GRA Taguchi & ANOVA. Int. J. Innov. Technol. Explor. Eng..

[4] Keblouti O., Boulanouar L., Aziz M., Yellese M. (2017). Effects of coating material and cutting parameters on the surface roughness and cutting forces in dry turning of AISI 52100 steel. Struct. Eng. Mech..

[5] Jindal P., Santhanam A., Schleinkofer U., Shuster A. (1999). Performance of PVD TiN, TiCN, and TiAlN coated cemented carbide tools in turning. Int. J. Refract. Met. Hard Mater..

[6] Fernández-Abia A., Barreiro J., Fernández-Larrinoa J., López de Lacalle L., Fernández-Valdivielso A., Pereira O. (2013). Behaviour of PVD coatings in the turning of austenitic stainless steels. Procedia Eng..

[7] Venkatesh V., Ye C., Quinto D., Hoy D. (1991). Performance Studies of Uncoated, CVD-Coated and PVD-Coated Carbides in Turning and Milling. CIRP Ann..

[8] Wang J. (2000). The effect of the multi-layer surface coating of carbide inserts on the cutting forces in turning operations. J. Mater. Process. Technol..

[9] Kulkarni A., Sargade V. (2015). Characterization and Performance of AlTiN, AlTiCrN, TiN/TiAlN PVD Coated Carbide Tools While Turning SS 304. Mater. Manuf. Process..

[10] Kamely M., Noordin M. (2011). The Impact of Cutting Tool Materials on Cutting Force. World Acad. Sci. Eng. Technol..

[11] Bach P., Trmal G., Zeman P., Vana J., Maly J. (2012). High Performance Titanium Milling at Low Cutting Speed. Proceedings of the 5th CIRP Conference on High Performance Cutting.

[12] Arrazola P., Özel T., Umbrello D., Davies M., Jawahir I. (2013). Recent advances in modelling of metal machining processes. CIRP Ann..

[13] Galanis N., Manolakos D. Finite Element Analysis of the Cutting Forces in Turning of Femoral Heads from AISI 316L Stainless Steel. Proceedings of the World Congress on Engineering 2014.

[14] Parihar R., Sahu R., Srinavasu G. (2017). Finite Element Analysis of Cutting Forces Generated in Turning Process using Deform 3D Software. Mater. Today Proc..

[15] Kumar S.C., Zeman P., Polcar T. (2020). A 2D finite element approach for predicting the machining performance of nanolayered TiAlCrN coating on WC-Co cutting tool during dry turning of AISI 1045 steel. Ceram. Int..

[16] Kara F., Aslantas K., Cicek A. (2015). ANN and multiple regression method-based modelling of cutting forces in orthogonal machining of AISI 316L stainless steel. Neural Comput. Appl..

[17] Qasim M. (2019). Prediction of Cutting Force in Turning Process by Using Artificial Neural Network. Al-Khwarizmi Eng. J..

[18] Mia M., Khan A., Dhar N. (2017). Study of surface roughness and cutting forces using ANN, RSM, and ANOVA in turning of Ti-6Al-4Vunder cryogenic jets applied at flank and rake faces of coated WC tool. Int. J. Adv. Manuf. Technol..

[19] Noordin M., Venkatesh V., Sharif S., Elting S., Abdullah A. (2004). Application of response surface methodology in describing the performance of coated carbide tools when turning AISI 1045 steel. J. Mater. Process. Technol..

[20] Kolar P., Fojtu P., Shmitz T. (2015). On Cutting force Coefficient Model with Respect to Tool Geometry and Tool Wear. Procedia Manuf..

[21] Shalaby M., El Hakim M., Veldhuis S., Dosbaeva G.K. (2017). An investigation into the behavior of the cutting forces in precision turning. Int. J. Adv. Manuf. Technol..

[22] Stachurski W., Midera S., Kruszynski B. (2012). Determination of Mathematical Formulae for the Cutting Force Fc during the Turning of C45 Steel. Mech. Mech. Eng..

[23] Mahmud S., Islam K., Habib A., Hossain S., Hoassain M. Optimization of Turning Parameters for Cutting Force and Chip Optimization of Turning Parameters for Cutting Force and Chip. Proceedings of the International Conference on Mechanical, Industrial and Materials Engineering 2015.

[24] Altintas Y. (2012). Manufacturing Automation: Metal Cutting Mechanics, Machine Tool.

[25] Cascóna I., Sarasuaa J. (2015). Mechanistic model for prediction of cutting forces in turning of non-axisymmetric parts. Procedia CIRP.

[26] Kienzle O. (1952). Die Bestimmung von Kräften und Leistungen an spanenden Werkzeugen und Werkzeugmaschinen. VDI-Z.

[27] Salehi M., Schmitz T., Copenhaver R., Haas R., Ovtcharova J. (2018). Probabilistic Prediction of Cutting and Ploughing Forces using Extended Kienzle Force model in Orthogonal Turning Process. Procedia CIRP.

[28] Popovic M., Tanovic L., Ehmann K. (2017). Cutting Forces Prediction: The Experimental Identification of Orthogonal Cutting Coefficients. FME Trans..

[29] Bera T., Manikandan H., Bansal A., Nema D. (2018). A Method to Determine Cutting Force Coefficients in Turning Using Mechanistic Approach. Int. J. Mater. Mech. Manuf..

[30] Horváth R., Lukács J. (2017). Application of a Force Model Adapted for the Precise Turning of Various Metallic Materials. J. Mech. Eng..

[31] Kovalčík J., Zeman P., Holešovský F., Mádl J., Kučerová L. (2020). Cutting force modelling with effects of cutting tool geometry and tool wear in milling of DIN C45 steel. MM Sci. J..

[32] Bartoszuk M., Grzesik W. (2015). Investigation of initial wear period of differently coated carbide cutting tools. J. Mach. Eng..

